# Exploring the Relationship between Semantics and Space

**DOI:** 10.1371/journal.pone.0005319

**Published:** 2009-04-27

**Authors:** Patrizia Turriziani, Massimiliano Oliveri, Sonia Bonnì, Giacomo Koch, Daniela Smirni, Lisa Cipolotti

**Affiliations:** 1 Dipartimento di Psicologia, Università di Palermo, Palermo, Italy; 2 C.I.R.A. Università di Palermo, Palermo, Italy; 3 Fondazione “Santa Lucia” IRCCS, Roma, Italy; 4 Dipartimento di Neuroscienze, Università di Roma Tor Vergata, Rome, Italy; 5 Department of Neuropsychology, National Hospital for Neurology and Neursurgery, Queen Square, London, United Kingdom; University of Groningen, Netherlands

## Abstract

The asymmetric distribution of human spatial attention has been repeatedly documented in both patients and healthy controls. Biases in the distribution of attention and/or in the mental representation of space may also affect some aspects of language processing. We investigated whether biases in attention and/or mental representation of space affect semantic representations. In particular, we investigated whether semantic judgments could be modulated by the location in space where the semantic information was presented and the role of the left and right parietal cortices in this task. Healthy subjects were presented with three pictures arranged horizontally (one middle and two outer pictures) of items belonging to the same semantic category. Subjects were asked to indicate the spatial position in which the semantic distance between the outer and middle pictures was smaller. Subjects systematically overestimated the semantic distance of items presented in the right side of space. We explored the neural correlates underpinning this bias using rTMS over the left and right parietal cortex. rTMS of the left parietal cortex selectively reduced this rightward bias. Our findings suggest the existence of an attentional and/or mental representational bias in semantic judgments, similar to that observed for the processing of space and numbers. Spatial manipulation of semantic material results in the activation of specialised attentional resources located in the left hemisphere.

## Introduction

The asymmetrical nature of the cognitive and neural mechanisms that underlie the distribution of attention and/or mental representations when processing space has been documented in studies of healthy controls and neurological patients. Typically, healthy controls show a leftward bias in perceptual tasks requiring, for example, the bisection of physical lines (for a review see [1]).

This leftward bias, termed pseudoneglect has also been reported in tasks involving numbers. Numbers are thought have a left to right representation. When comparing numerical intervals, normal subjects overestimate the difference between the middle number and the outer number positioned at its left side [2], [3]. Pseudoneglect has been attributed to a specific attentional and/or representational bias towards the left hemi-space

Spatial biases have been reported also in neurological patients with lesions and spatial neglect syndrome. This syndrome is often associated to right hemisphere lesions. Typically, patients tend to neglect the left hemi-space in perceptual tasks, including physical line bisection [4] and representational tasks, such as the description of a familiar place from opposite view-points from memory [5]. Neglect has been documented also in numerical processing tasks [6–9, but see 10]. For example, neglect patients display a rightward bias when asked to “bisect” a mental number line tend to neglect numbers on the left.

The left hemispace neglect has been interpreted by different models regarding the spatial distribution of attention [4], [11]. To consider the Kinsbourne's and Heilman's models. According to the Kinsbourne's model, the “rightward attentional vector” of the left hemisphere dominates the “leftward attentional vector” of the right hemisphere. Following this, lesions of the right hemisphere would result in an increase the rightward bias of spatial attention due to the impairment of the reciprocal inhibition coming from this hemisphere [12]. The Heilman's model postulated that the right hemisphere is dominant in the distribution of attention for both hemifields, while the left hemisphere attentional vector is directed only to the right hemifield. Therefore, right hemisphere lesions result in a more frequent contralesional attentional impairment than left hemisphere lesions [13]. This model assumes that spatial neglect is a deficit of attention in the left- rather than an increase of attention in the right space.

Interestingly, recent studies have suggested the some verbal domains, such as alphabetical strings, with left to right representation, may also have a spatial organisation. Healthy participants showed a leftward bias when they were shown three-letter strings, and asked to estimate which of the two flankers (e.g. C and P) was of greater alphabetical distance from the inner-letter (H). This finding has been interpreted as demonstrating an attentional and/or representational bias towards letters located on the left-hand side of the mental alphabetical line [14]–[15]. Interestingly, patients with neglect tend to omit letters presented on the left side of the mental representation line, revealing the same rightward bias in the letter bisection tasks as in the physical line bisection tasks [16].

Overall, the attentional and/or representational bias favouring the left hemi-space that has been observed for space and numbers suggests the preferential involvement of right hemisphere structures. In line with this, neuroimaging and lesion studies have implicated the involvement of a right fronto-parietal network in both pseudoneglect and neglect [17]–[19]. In particular, activation of the right posterior parietal cortex has been associated with bisection tasks involving both physical and mental number lines [20]–[22].

It remains unclear whether the reported biases for space, numbers and alphabetical strings are observed also for linguistic components without a left to right. For example, studies investigating the processing of letters that were not organised alphabetically have yielded conflicting results. Some studies found that when participants were required to bisect letter lines, they showed a bias toward the left hemi-space [23]–[25]. In contrast, Lee and colleagues [26], [27] documented a systematic bias towards the rightward bisection of letter lines in both healthy controls and neglect patients. The authors proposed that the verbal information available may be an important determinant of the direction of bisection errors. They suggested that the observed rightward bias was the result of activation of left language areas.

Recently, Mohr and Leonard [28] investigated the impact of semantic information on letter line bisection in healthy subjects. They used letter lines with embedded words that were either emotional (e.g. eucsoiaadkillfp) or neutral (e.g. aheaiinebmainul). The results revealed a stronger rightward bisection bias for letter lines containing emotional words. The authors thus suggested that semantic information may modulate performance on a bisection task. They argued that the semantic information activated the left hemisphere more strongly than the right hemisphere, and thus led to a rightward shift of attention away from the actual centre of the letter line [29], [30].

Interestingly, a recent fMRI study investigated the ability to orient attention to the semantic categories of words using a cued (semantic or spatial) lexical decision task [31], similar in structure to the Posner attentional orienting task. The results showed that semantic orienting selectively activated left fronto-parietal brain areas that are known to be involved in the semantic analysis of word stimuli.

To the best of our knowledge, the issue of whether the attentional and/or representational bias affects semantic processing tasks has not been investigated. However, it has been proposed that some spatial factors are involved in the representation of meaning. Zannino and colleagues [32] provided normative data for the featural representation of concepts. They adopted a metric representation of the relationship between categories, and according to it calculated a measure of semantic distance, which evaluated the degree of semantic similarity between concepts. The semantic distance between pairs of concepts was represented in the form of a vector, which had as many positions as the overall amount of unique features generated by control subjects when asked to define a specific concept (e.g., “has legs” or “used for making cakes”). This study coincided with models that assume that semantic information is organised within a semantic space [33]–[36]. According to these models, concepts that are highly semantically associated are in close spatial proximity to each other, whereas weakly associated or un-associated concepts are far from each other. The performance of healthy participants has been shown to be faster and more accurate when judging concepts that are semantically distant than concepts that are semantically close [32], [36]. This phenomenon is similar to the distance effect observed with numbers [37], whereby the time required to compare the magnitude of two numbers decreases as the distance between them increases.

The aim of our study was to investigate for the first time whether spatial variables, such as the spatial position (left vs. right) in which semantic information is presented, modulate the performance of healthy participants in a semantic judgment task. Moreover, we aimed to explore the role of left versus right posterior parietal cortices in this task by using repetitive transcranial magnetic stimulation (rTMS). Previous research has demonstrated that stimulation of the right posterior parietal cortex causes a rightward shift in the bisection of physical lines [38] and in mental number bisection [2], [3]. Stimulation of the homologous regions in the left hemisphere had no effect [2], [3].

## Methods

Four behavioural and one rTMS experiment were conducted.

### Participants

Fifty-height neurologically normal right-handed participants (12M, 35F; mean age: 25.2±3.7 years) participated in the study. All participants were native Italian speakers with normal or corrected to normal vision and all, except two, were naïve to the purpose of the study. Written and informed consent was obtained prior to testing, in accordance with local ethical committee regulations of the Fondazione Santa Lucia (Rome, Italy).

### Experiments 1 and 2: Semantic distance judgment task

We conducted two different experiments to investigate the relationship between the semantic distance between concepts and the location in which these concepts were presented.

#### Experiment 1

Ten subjects (1M, 9F; mean age: 25.4±3.5 years) participated in this experiment.

Sixty-four line drawings were selected from Snodgrass and Vanderwart's (45 items) and Dell'Acqua, Lotto, and Job's (19 items) batteries [39], [40]. These pictures were all previously used in a study by Zannino and colleagues [32]. The pictures comprised items from four semantic categories, including two categories of living things (16 fruits and 16 mammals) and two of non-living objects (16 pieces of furniture and 16 vehicles). For each of the 64 pictures, normative data for concept familiarity, age of acquisition, name agreement and visual complexity were available.

**Figure 1:**
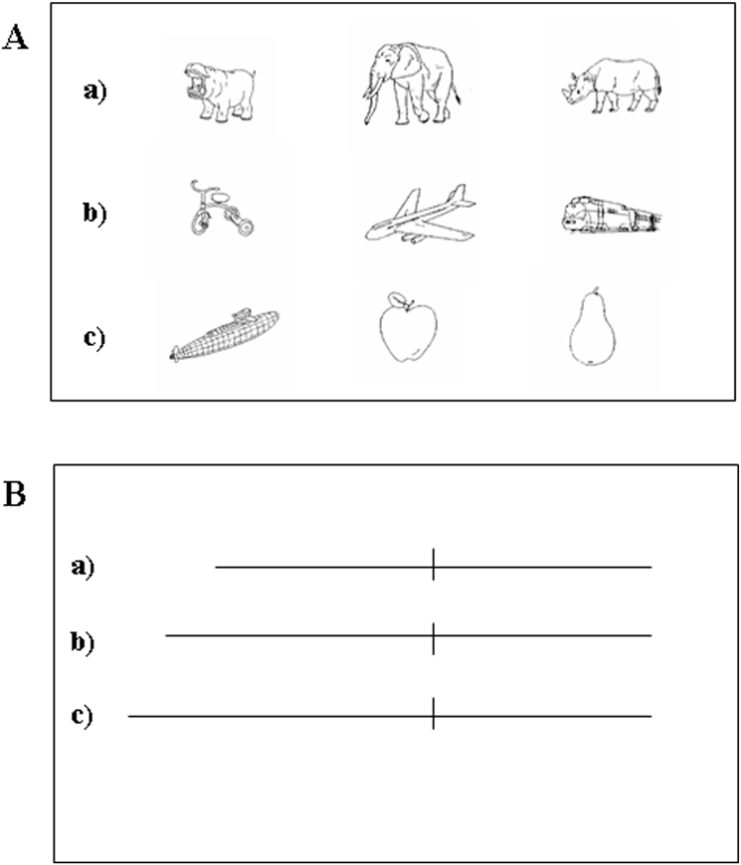
Schematic of the experimental design showing sample stimuli and experimental conditions. (A) Example stimuli of 1) Near within-category condition; 2) Same condition; 3) Near across-category condition in the Semantic distance judgment task; (B) Example stimuli of 1) Near within-category condition 2) Same condition 3) Near across-category condition in the Spatial judgment task.

These 64 pictures were combined to obtain 180 different triplets. Each triplet consisted of one middle and two outer pictures. Size for each picture was 4°×3°. The two outer pictures were presented with 5° of eccentricity to the left and right of the middle picture (see Figure 1A).

For each triplet, an index expressing the semantic distance between the middle picture and each of the outer pictures was calculated using the normative data reported by Zannino at al. [32]. These authors established vectors that can be used to compute the semantic distance between two concepts. Using these vectors, a chi-square value was computed to represent the semantic distance between any two pictures. Furthermore, we adopted the method developed by Zannino to calculate an index of semantic distance for each triplet of items used in our semantic task (this file can be downloaded from www.hsantalucia.it). In the present study, the value for the semantic distance was calculated by comparing the vector of the middle item in each triplet with each of the corresponding outer items. Thus, within each semantic category, semantic distance indices ranging from 9.42 to 18.44 were available for each of the two pairs of concepts that constituted a triplet (e.g. pear apple pineapple; pear-apple = 10.46; apple-pineapple = 15.52).

There were three experimental conditions: Near within-category, Same, and Near across-category (Figure 1A). The semantic category of a triplet was always defined by the middle item. In the Near within-category condition, the triplets belonged to the same semantic category, but the semantic distance between the middle and the outer pictures was smaller for one of the outer pictures (e.g. train, aeroplane and tricycle). In the Same condition, triplets were composed of three members of the same semantic category with identical semantic distance between the middle and the two outer pictures (e.g. hippopotamus, elephant, rhinoceros). In the Near across-category condition, one outer item in each triplet belonged to a different semantic category (e.g. apple, pear and submarine). In this condition, the items that were semantically related (e.g. pear-apples) had a smaller semantic distance between them than the semantically unrelated items within the triplet (e.g. pear-submarine).

There were 60 triplets in each of the three experimental conditions.

#### Procedure

Before starting the experiment, participants were asked to name the 64 pictures.

Triplets were presented for 1000 ms on a 19-inch computer monitor. The middle picture was always presented in the centre of the monitor.

The intertrial interval was 2500 ms. Participants were seated at a distance of 45 cm from the monitor and were asked to focus on a central fixation cross that preceded item presentation.

Participants were asked to indicate the side of space in which the semantic distance between the outer and middle pictures was smallest (“where is the picture that is semantically closest to the middle picture?”). Participants responded by pressing one of three buttons with their right middle, index or ring finger for “same,” “left” or “right” responses, respectively. Participants were told to choose the “same” response if neither of the two outer pictures appeared to be more semantically related to the middle item. The side of space in which the target picture appeared within each triplet was randomised.

#### Results

Accuracy (mean number of errors) and reaction times (RTs: interval of time between the onset of stimuli and the participant's response) were analysed. Only RTs for correct responses were analysed. We performed a repeated measures ANOVA on the mean number of errors, with Condition (Same, Near within-category, Near across-category) and Space (left, right) as within-subjects factors. Planned comparisons of single factors were only carried out with significant group factors.

**Figure 2:**
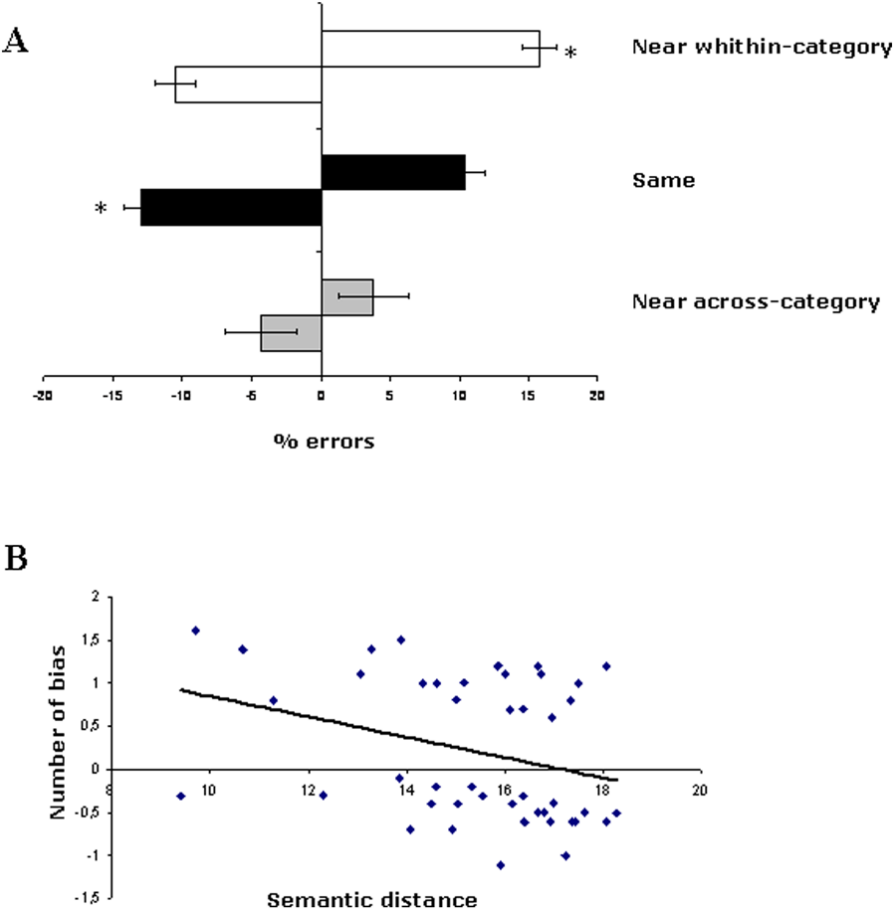
Experiment 1: Semantic distance judgment task. (A) Mean leftward and rightward errors (±1 SE) as a function of the different experimental conditions. Negative values indicate leftward shifts and positive values rightward shifts in the judgment; (B) Bias in this experiment as a function of the semantic distance in the semantic distance judgment task. Rightward bias decreased as the magnitude of the semantic distance increased.

We conducted three separate analyses. The first analysis investigated the effect of semantic distance within and between categories and their location in space. A 3x2 repeated measures ANOVA was conducted on mean errors with the variables of Condition (Near within-category×Same×Near across-category) and Space (left×right). As shown in Figure 2A, there was a significant main effect of Condition [F (2,18) = 6.9; p<0.005], with lowest error rates observed for the Near across-category condition. The average number of errors in the Near across-category condition was significantly different from both the Same [F (1,9) = 5.5; p<0.04] and Near within-category [F (1,9) = 21.8; p<0.001] conditions. The error rates in the Same and Near within-category conditions were comparable [F (1,9) = .22; p>0.5]. The main effect of Space was not significant [F (1,9) = 1.8; p>0.5], however, the interaction of Condition×Space was significant [F (2,18) = 6.16; p<0.005]. To investigate this interaction we conducted planned comparisons. We found a rightward bias in the Near within-category condition. Specifically, in trials where the semantic distance was smaller between the middle and the right picture, participants tended to produce erroneous “left” or “same” responses [F (1,9) = 7.9; p<0.05]. In the Same condition, on the other hand, a leftward bias was found. Participants erroneously judged the semantic distance between the middle and the left picture to be smaller [F (1,9) = 8.8; p<0.05]. There was no significant difference between leftward and rightward biases in the Near across-category condition [F (1,9) = .28; p>0.5].

The second analysis investigated the leftward and rightward biases in all experimental conditions. In the Near within-category and Near across-category condition, the leftward bias was calculated as the average of “right” and “same” responses when the semantic distance was actually smaller in the left side of space. The rightward bias was the average of “left” and “same” responses when the semantic distance was actually smaller in the right side of space. Negative values were assigned to leftward shifts and positive values to rightward shifts in judgment. An ANOVA was conducted with the variables Condition (Near within-category×Same×Near across-category) and Bias (leftward×rightward). The analysis revealed a main effect of Condition [F (2,18) = 2,57; p<0.05], with lowest bias observed for the Near across-category condition, different from both the Same [F (1,9) = 5.5; p<0.04] and Near within-category [F (1,9) = 89,16; p<0.001] conditions. The bias in the Same and Near within-category conditions was comparable [F (1,9) = 1,9; p>0.5]. The main effect of Bias was also significant [F (1,9) = 128,63; p>0.000], revealing a rightward bias. The interaction of Condition×Bias was also significant [F (2,18) = 121,32; p<0.005]. Planned comparisons showed a rightward bias in the Near within-category condition [F (1,9) = 333,79; p<0.000] and in the Same condition [F (1,9) = 7,66; p<0.02]. There was no significant difference between leftward and rightward biases in the Near across-category condition [F (1,9) = 1,2; p>0.5].

The third analysis investigated whether the degree of rightward bias in the Near within-category condition varied as a function of semantic distance. We calculated the average bias across subjects for each triplet, and correlated this value with the value of the semantic distance between the two semantically-close words using the Pearson's correlation coefficient test. There was a negative correlation (r = −.31; p<.05) between the semantic distance and the average rightward bias. This result suggests that the rightward bias was modulated by the value of the semantic distance between the middle picture and the semantically-closer outer picture in each triplet. Specifically, the rightward bias was greater when the semantic distance between the concepts was smaller (Figure 2B).

The ANOVA performed on the RT data did not reveal any significant differences between the left- and right location of the target outer picture of the Near within-category conditions [F (1,9) = .36; p>0.5]. Moreover, there was no significant difference in RT when comparing left and right Near across-category conditions [F (1,9) = .38; p>0.5]. However, RTs were significantly slower in the Same than in the Near across-category conditions [F (2,18) = 5.52; P<0.05]. They were modulated by task difficulty (semantic distance was easier to judge with triplets of the Near across-category condition), but not by the spatial location of the semantically-close outer picture.

In summary, semantic judgments were influenced by the spatial location of the stimuli. When comparing the semantic distance between pairs of pictures, healthy participants tended to overestimate the distance between the middle reference picture and the outer picture that was positioned to its right.

#### Experiment 2

The aim of this experiment was to replicate and extend the results obtained in Experiment 1. Participants were ten right-handed normal volunteers (2M, 8F; mean age: 25±2.9 years). The stimuli and procedure were the same as in Experiment 1 but instructions differed. Participants were asked to indicate the side of space where the semantic distance between the outer and middle pictures was greater (“where is the picture that is most semantically different to the middle picture?”). Participants responded as previously with their right middle, index or ring finger for “same,” “left” or “right” responses, respectively.

#### Results

**Figure 3:**
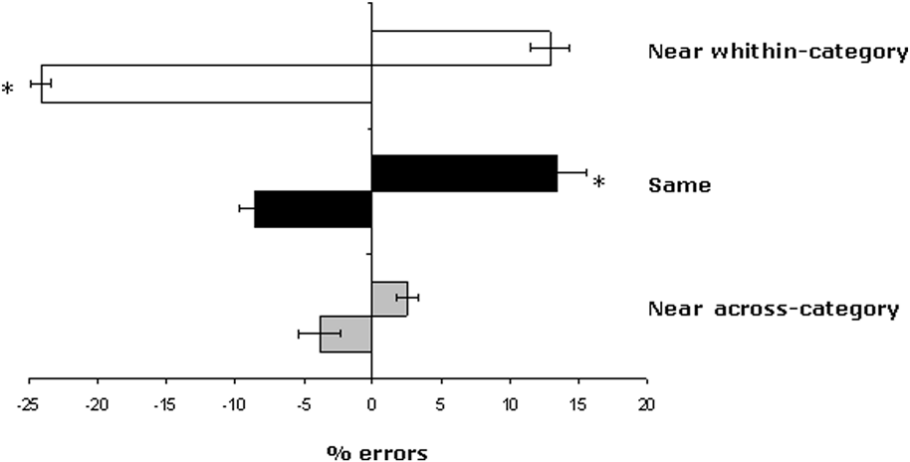
Experiment 2: Semantic distance judgment task. Mean leftward and rightward errors (±1 SE) as a function of the different experimental conditions. Negative values indicate leftward shifts and positive values rightward shifts in the judgment.

The same 3×2 repeated measures ANOVA used in Experiment 1, with the variables of condition (Near within-category×Same×Near across-category) and Space (left×right) was conducted. Figure 3 shows the results of this experiment. The ANOVA on mean errors revealed a significant main effect of Condition [F (2,18) = 39.61; p<0.001], with the lowest error rates observed in the Near across-category condition, as in Experiment 1. The mean number of errors on the Near across-category condition was significantly different from both the Same [F (1,9) = 15.5; p<0.005] and the Near within-category [F (1,9) = 4.9; p>0.05] conditions. The main effect of Space was not significant [F (1,9) = 4.61; p>0.05]. However, the interaction of Condition×Space was significant [F (2,18) = 31.32; p<0.001]. To investigate this interaction we conducted planned comparisons, which revealed a rightward bias in the Near within-category and Same category conditions [F (1,9) = 47.9; p<0.0001, F (1,9) = 6.4; p<0.05, respectively]. In other words, in trials where the semantic distance was greatest between the middle and left pictures, participants erroneously indicated either that the greater semantic distance was that between the middle and the right picture, or that the semantic distance between the two picture pairs was identical. There was no significant difference between leftward or rightward biases in the Near across-category condition [F (1,9) = .82; p>0.5].

An ANOVA performed on RT data revealed an identical pattern of results as in Experiment 1.

In summary, when asked to indicate which side of space contained the picture that was of greater semantic distance from the middle reference picture, the performance of healthy participants nevertheless showed a rightward bias. This pattern of results replicated and extended those obtained in Experiment 1, suggesting that the semantic distance between the middle and the right outer pictures tends to be overestimated, regardless of the type of judgment being made.

### Experiment 3: Eye movements and semantic distance judgment task

During the semantic distance judgment tasks, subjects had to make semantic judgments based on three pictures simultaneously presented in a relatively large time. The participants were instructed to always fixate centrally, however it is possible that they shifted their gaze between pictures. The aim of this experiment was to investigate whether semantic judgments were associated to overt or covert spatial attention shifts. To address this point, we measured subjects'eye movements during the semantic distance judgment task used in Experiment 1.

Participants were six right-handed normal volunteers (4M, 2F; mean age: 28±2.1 years). None of them participated in Experiment 1. The stimuli and procedure were the same as in Experiment 1. Eye position was assessed trough electrooculogram (EOG). EOG responses were recorded by means of Ag/AgCl surface electrodes producing binocular horizontal EOG. The signal was amplified and bandpass filtered (5–2000 Hz) using SIGNAL software. The upward deflection of the recorded eye position signal in EOG channels corresponded to rightward eye movement. EOG was used to assess saccades and initial saccadic latencies.

#### Results

Mean saccadic latencies were analyzed for each experimental condition during the task, using a 3×2 repeated measures ANOVA with the variables of Condition (Near within-category×Same×Near across-category) and Direction of saccades (leftward×rightward).

In the trials with errors, saccadic latencies were comparable in all experimental conditions [Condition main effect: F (2,10) = 3,69; p>0.5]. Saccadic latencies were slower in the right than in the left space [Direction main effect: F (1,5) = 20,00; p<0.006]. The interaction of Condition×Direction was not significant [F (2,10) = 1.95; p<0.19].

In the trials with correct responses, the ANOVA always did not show significant main effects of Condition [F (2,10) = 2,23; p<0.15] and Direction [F (1,5) = 3,56; p<0.11]. However, the interaction of Condition×Direction was significant [F (2,10) = 4,5; p<0.05]. Planned comparisons revealed that rightward latencies were slower than leftward [F (1,5) = 5,00; p<0.05] in the Near within-category condition. There was no significant difference between leftward and rightward biases in the Same [F (1,5) = 0,55; p>0.5] and in the Near across-category [F (1,5) = 1,8; p>0.5] conditions.

Thereafter, the ratio of the number of leftward/rightward saccades was analyzed for each experimental condition during the task, using a 3×2 repeated measures ANOVA with the variables Condition (Near within-category×Same×Near across-category) and Direction of saccades (leftward×rightward).

In the trials with errors, there was not any significant main effect of Condition [F (2,10) = 3,16; p>0.05]. The direction of saccades was comparable within all conditions. The Direction main effect was significant [F (1,5) = 10,89; p<0.02], showing a greater number of rightward saccades. The interaction Condition×Direction was also significant [F (1,5) = 5,00; p<0.05]. Planned comparisons revealed greater leftward saccades in the Same [F (1,5) = 6,73; p<0.05] and in the Near across-category [F (1,5) = 6,01; p<0.05] conditions. There was no difference between leftward and rightward saccades in the Near within-category condition [F (1,5) = 4,00; p>0.1].

In the trials with correct responses, there was a significant main effect of Direction [F (1,5) = 8,43; p<0.05], with greater rightward than leftward saccades. However, there was not any significance of the Condition [F (1,5) = 0,65; p>0.4] effect, nor an interaction between the two factors [F (1,5) = 4,69; p>0.05].

In summary, our semantic judgment tasks involved overt saccades. The pattern of latencies and direction of saccades were similar in trials with errors and in trials with correct responses. The number of rightward saccades was higher than the leftward ones; the latencies of rightward saccades were slower than the leftward ones.

### Experiment 4: Spatial (physical line) judgment task

The aim of this experiment was to evaluate the performance of healthy participants in a pre-bisected line judgment task. The present experiment examined whether the direction of the attentional bias demonstrated above is also observed in tasks involving language and physical space.

Eleven participants (3M, 8F; mean age: 25±2.9 years) took part in this experiment.

Stimuli consisted of 120 different pairs of pre-bisected lines. Each line consisted of two segments measuring 70 mm, 75 mm, or 80mm long. The relative length of the two segments varied across trials, being either equal or shorter on one side of space (see Figure 1B).

There were three experimental conditions: Same, Near within-category and Near across-category. In the Near within-category condition, trials were composed of asymmetrically bisected lines, with two segments of 70 and 75 mm each (i.e. either the left or the right segment was 5 mm shorter). In the Same condition, trials consisted of symmetrically bisected lines, with two equal segments of 70 mm each. In the Near across-category condition, trials were composed of asymmetrically bisected lines, with two segments of 70 and 80 mm each (i.e. either the left or the right segment was 10 mm shorter).

Each experimental condition was made up of 40 trials.

#### Procedure

Before starting the experiment, a practice trial was administered to ensure the participant's confidence in the task.

Stimuli were presented for 50 ms each, with the bisector of each line always positioned in the centre of the monitor. The intertrial interval was 2500 ms.

Participants were asked to indicate the side of space in which the shorter line segment was located (“where is the shorter line?”). They responded by pressing one of three buttons with their right middle, index or ring finger for “same,” “left,” or “right” responses, respectively. The side of space where the target segment appeared in the Near-across and Near-within category conditions was randomised.

#### Results

Accuracy and reaction times were analysed. Only RTs for correct responses were analysed. We performed a repeated measures ANOVA on the mean number of errors, with Condition (Same, Near within-category, Near across-category) and Space (left, right) as within-subjects factors.

**Figure 4:**
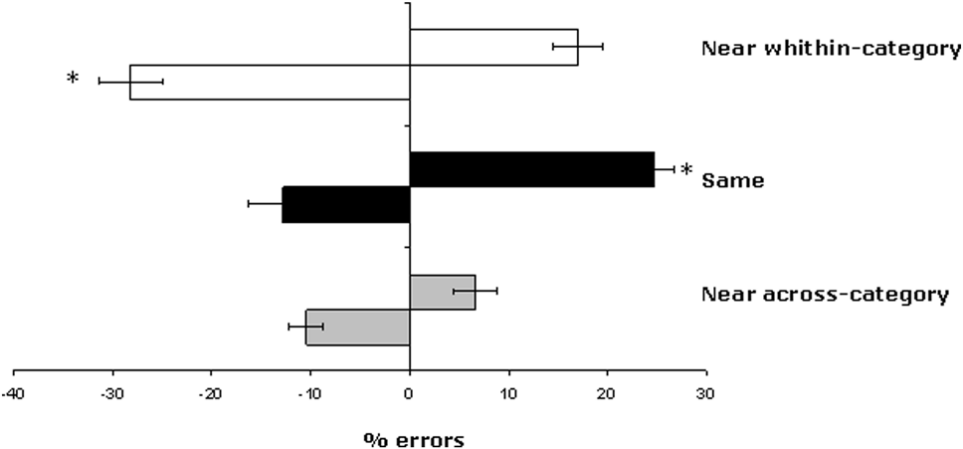
Experiment 4: Spatial judgment task. Mean leftward and rightward errors (±1 SE) as a function of the different experimental conditions. Negative values indicate leftward shifts and positive values rightward shifts in the judgment.

Figure 4 shows that spatial judgments were influenced by the spatial location in which the shorter line segment appeared. A 3×2 repeated measures ANOVA, with the variables of condition (Near within-category×Same×Near across-category) and Space (left×right) was conducted. There was a significant main effect of Condition [F (2,20) = 11.54; p>0.005], with lowest error rates observed in the Near across-category condition. The mean number of errors in the Near across-category condition was significantly different from both the Same [F (1,10) = 13.12; p<0.005] and the Near within-category [F (1,10) = 59.17; p>0.001] conditions. The error rates in both the Same and the Near within-category conditions were comparable [F (1,10) = .92; p<0.5]. Analyses did not yield a significant main effect of Space [F (1,10) = .23; p>0.5]. However, the Condition×Space interaction was significant [F (2,20) = 14.31; p<0.001]. A planned comparison of this interaction revealed a leftward bias in the Near within-category condition. In other words, participants showed a tendency to erroneously indicate that the shorter segment was in the right side of space [F (1,10) = 10.98; p<0.01]. Similarly, in the Same condition, where the two segments were identical, participants produced incorrect responses by indicating that the shorter line segment was located in the right side of space [F (1,10) = 9.5; p<0.01]. There was no significant difference between leftward and rightward biases in the Near across-category condition [F (1,10) = 2.91; P>0.5].

The analysis of RT data did not reveal any significant effects. An ANOVA found no significant differences between the Same, left-, and right- Near within-category conditions [F (2,20) = 1.97; p>0.5], nor between the Same, left- and right-side Near across-category conditions [F (2,20) = 3.11; p<0.5].

### Experiment 5: Neural correlates of a semantic distance task

The aim of this experiment was to investigate the neural correlates of the semantic distance judgement task used in Experiment 1.

Twenty-one right-handed normal volunteers (4M, 16F; mean age: 21±2.2 years) participated in this experiment.

#### Procedure

We used repetitive transcranial magnetic stimulation (rTMS) trains at frequencies known to transiently inhibit the neural activity of a cortical area during the execution of a cognitive task. rTMS was applied over P3 and P4 of the 10–20 EEG system of healthy participants performing the semantic distance task. Posterior parietal cortices have previously been linked to “space tests”, namely tasks requiring an interaction between space and numbers [9] or time [41].

A MagStim Super Rapid magnetic stimulator (Whitland, UK), able to deliver trains at a maximum frequency of 50 Hz, was used. The stimulator was connected to a focal 70 mm coil, to minimize discomfort from oral-facial muscle movement. For each participant, single pulse TMS was then applied at decreasing intensities to determine the motor threshold, which was defined as the minimal TMS intensity capable of inducing a reliable muscle twitch in the contralateral hand on 50% of trials within a sequence of ten consecutive trials.

For each scalp site, an rTMS train of a10 min duration and 1 Hz frequency ( = 600 stimuli) was applied at an intensity of 90% of the motor threshold. The experiment was conducted in a soundproof, dimly lit room. Participants sat comfortably on an armchair, at a distance of 50 cm from a computer monitor, which was placed so that its centre was at the participant's eye-level.

Participants were randomly allocated into one of two groups. One group (10 subjects) of performed the semantic distance judgment task following rTMS over the left parietal cortex, whereas the other group (11 subjects) performed this same task following rTMS over the right parietal cortex. The semantic distance judgment task immediately followed the rTMS trains.

The performance of the groups following rTMS of the right and left posterior parietal cortex was separately compared with that of the participants tested in Experiment 1, who were not administered rTMS (baseline).

#### Results

Accuracy and reaction times were analysed. Only RTs for correct responses were analysed. We performed a repeated measures ANOVA on the mean number of errors, with Condition (Same, Near within-category, Near across-category) and Space (left, right) as within-subjects factors. Factor of Session (left parietal rTMS, right parietal rTMS, baseline) was also analysed using a mixed factorial design.

**Figure 5:**
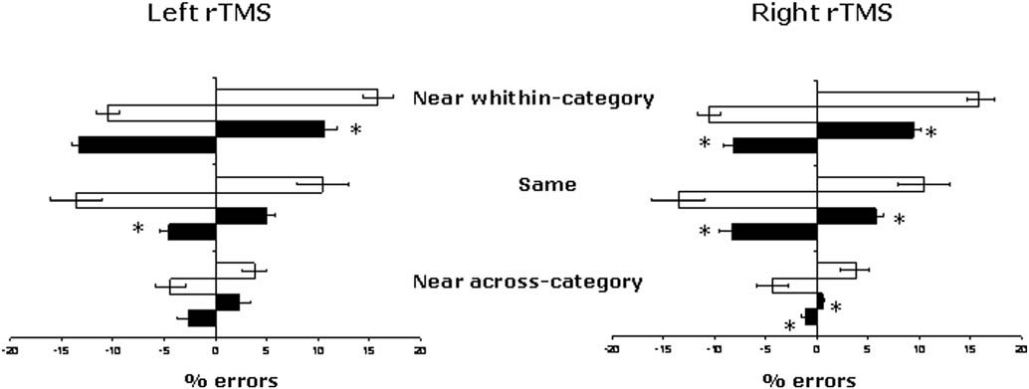
Experiment 5: Mean leftward and rightward errors (±1 SE) in the Semantic distance judgment task in the different experimental conditions. Negative values (black bars) indicate leftward shifts and positive values (white bars) rightward shifts in the judgment. White bars indicate leftward and rightward errors in baseline. Black bars indicate leftward and rightward errors in left rTMS (left panel) and right rTMS (right panel) sessions. rTMS = repetitive transcranial magnetic stimulation.

Figure 5 shows the mean percentage of errors in the Near within-category, Same and Near across-category conditions following left and right rTMS.

To analyse the effect of left and right parietal rTMS, a mixed factorial ANOVA was conducted to compare the performance of the different groups (left rTMS vs. baseline; right rTMS vs. baseline; left rTMS vs. right rTMS).

A 3×2×2 mixed factorial ANOVA, with the variables of Condition (Near within-category×Same×Near across-category), Space (left×right), and Session (left rTMS×baseline) revealed a significant main effect of Session [F (1,19) = 14.88; p<0.001]: Participants who received rTMS over the right parietal cortex made significantly fewer errors compared with the controls from Experiment 1 who did not receive rTMS. There was also a significant main effect of Condition [F (2,38) = 22.07; p<.000], with fewer errors observed in the Near across-category condition compared with both the Same [F (1,19) = 8.72; p<0.008] and the Near within-category [F (1,19) = 73.25; p<0.0000] conditions. The interaction of Condition×Space×Session was also significant [F (2,38) = 11.21; p<0.0001]. In the Near-within category condition, rTMS applied over the left parietal cortex reduced the frequency of rightward errors [F (1,19) = 7.55; p<0.01] and increased that of leftward errors [F (1,19) = 4.71; p<0.04]. In the Same condition, left parietal rTMS reduced the number of leftward errors [F (1,19) = 12.18; p<0.002]. In the Near across-category condition, left parietal rTMS did not specifically affect any side [F (1,19) = .03; p>0.5]. All other effects and interactions were not significant.

A 3×2×2 mixed factorial ANOVA with the variables of Condition (Near within-category×Same×Near across-category, Space (left×right), and Session (right rTMS×baseline) also revealed a significant main effect of Session [F (1,18) = 29.85; p<0.000]: the group who received right rTMS made fewer erroneous judgments compared with the baseline group (Experiment 1). A further main effect of Condition was also found [F (2,36) = 19.83; p<0.000], with lower error rates in the Near across-category condition compared with the Same [F (1,18) = 16.27; p<0.007] and the Near within-category [F (1,18) = 67.33; p<0.000] conditions. In contrast with the previous analysis, the interaction of Condition×Space×Session was not significant [F (2,36) = 2.33; p = 0.111]. All other effects and interactions were also not significant.

The effects of rTMS on the semantic distance judgment task were assessed using a 3×2×2 mixed factorial ANOVA, with the variables of Condition (Near within-category×Same×Near across-category), Space (Left×Right), and Session (left rTMS vs. right rTMS). The results revealed that both left and right rTMS affected the leftward number of errors in both the Near within-category [F (1,19) = 17.8; p<0.0004] and the Same [F (1,19) = 7.03; p<0.01] conditions. As statistical analysis described above, left rTMS increase leftward errors whereas right rTMS decrease leftward errors.

**Figure 6:**
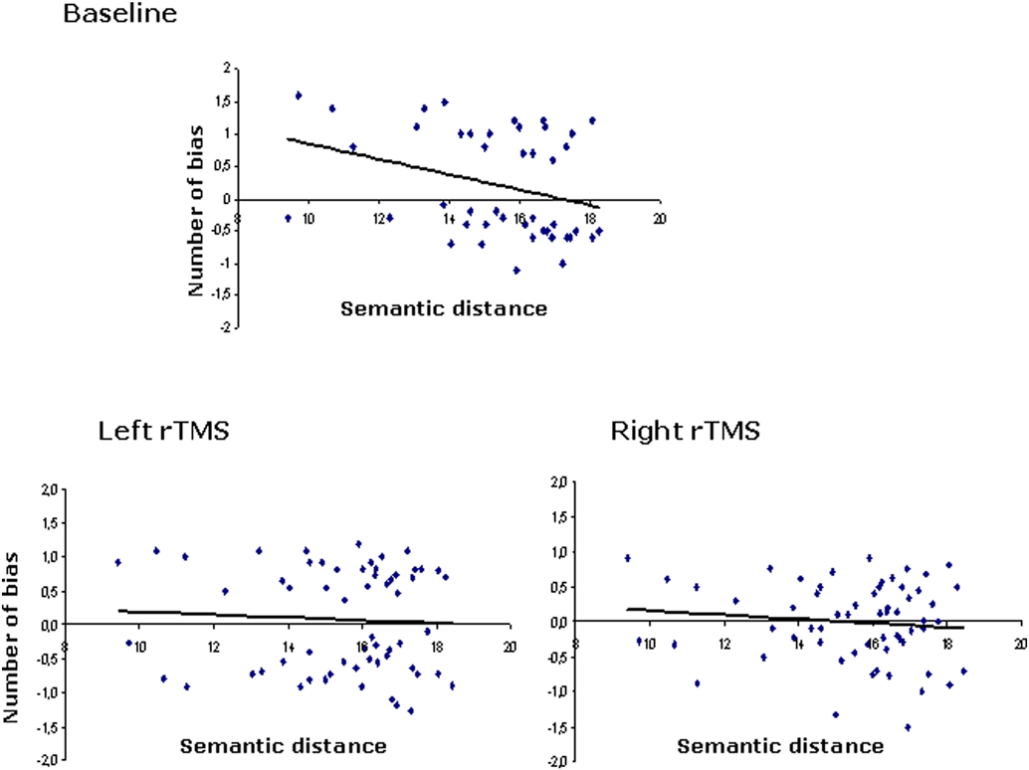
Bias in Experiment 1 (top panel) and Experiment 5 (bottom panel) as a function of semantic distance. In Experiment 1, rightward bias in the Semantic distance judgment task decreased as the magnitude of the semantic distance increased (top panel); in Experiment 5 (bottom panel), left parietal rTMS disrupted the correlation between rightward bias and semantic distance, whereas right parietal rTMS did not affect this correlation.

Following the same method used in Experiment 1 to assess the correlation between leftward and rightward biases and the value of the semantic distance between the stimuli, further analyses were conducted to explore whether left and right parietal rTMS affected this correlation (Figure 6). A Pearson's correlation coefficient test revealed a negative correlation (r = −.11; p<.05) between semantic distance and rightward bias following right parietal rTMS. Conversely, left parietal rTMS disrupted the negative correlation between rightward bias and semantic distance showed in the baseline session (r = −.066; p = .64). We also calculated the difference between the two correlation coefficients. The correlation coefficient for left parietal rTMS was different to that of the baseline session (p = .04); on the other hand, the correlation coefficient for right parietal rTMS did not differ from that of the baseline session (p = .13).

Analyses of RT data did not reveal any significant effects.

In summary, these findings indicate that left parietal rTMS disrupted the negative correlation between semantic distance and rightward bias in a semantic distance judgement task, whereas right parietal rTMS did not substantially alter performance on this task.

## Results and Discussion

In this paper we set out to investigate whether spatial variables, such as the spatial location in which semantic information is presented, modulate the performance of healthy participants in semantic judgments tasks. Moreover, we were interested in examining the role of the left and right parietal cortices in this task. We carried out four behavioural and one rTMS study.

The two main findings of our present study were: 1) Participants showed a rightward bias in a bisection task involving semantic distances. In contrast they showed a leftward bias observed in a physical line bisection task. The analysis of the eye movement suggests that the rightward bias in the semantic judgement task is linked to overt shift of attention. However, the pattern of latencies and direction of saccades were similar in trials with errors and correct responses. This suggest that the eye movements are not solely responsible for the reported rightward bias in trials with errors; 2) Interestingly, the rightward bias was significantly diminished following left, but not right, parietal cortex rTMS.

To discuss first the behavioural evidence from the semantic and spatial judgment tasks. In both Experiments 1 and 2, we showed that when asked to compare the semantic distance between concepts, healthy participants consistently overestimated semantic distances in the right side of space. This bias was observed regardless of whether participants had to judge which side of space contained the picture that was semantically closer (Experiment 1) or more distant (Experiment 2) to a middle reference picture. This demonstrates that task instructions and motor responses could not account for the bias. Moreover, we found that bisection errors in a physical line bisection task had a bias towards the left side of space (Experiment 4). This suggests that our observed rightward bias was specific for the processing of verbal material.

Interestingly, we found that the magnitude of the semantic distance had an effect on the bias. A negative correlation was documented between the extent of the bias deviation and the semantic distance. Rightward bias increased as semantic distance decreased, suggesting that semantically close concepts were more likely to have their distance underestimated. Interestingly, semantic distance also modulated reaction times. We found that participants were faster when asked to judge triplets that contained items belonging to different semantic categories. However, their reaction times were not influenced by the spatial location of the semantically-related picture.

Our findings suggesting that spatial location affects performance on semantic processing are in line with recent findings that the emotional connotation of words increases rightward bias in a letter line bisection task [28]. In this context, we note that there are at least three lesion studies suggesting a link between space and language. Coslett reported that in some aphasics, the direction in which they orient their attention influences their use of language [42]. He investigated an aphasic patient who was poorer at understanding spoken language and producing words when attending to his right than to his left hemispace. Chatterjee et al. [43] described an agrammatic patient whose production and comprehension of sentences was influenced by spatial factors. Rinaldi and co-workers [44], [45] reported that patients with neglect made significantly more errors when asked to compare two spoken sentences if the emphatic stress was placed at the beginning. These results seem to support the Coslett's “Spatial Registration Hypothesis” [42], suggesting that each perceived stimulus is automatically marked with reference to its co-ordinates in egocentric space, even if spatial information does not seem relevant for the task at hand.

Turning now to discuss the results of our TMS experiment. We found that left parietal rTMS disrupted the rightward bias, while right parietal rTMS had no effect on this bias. Moreover, we found that left parietal rTMS modified the negative correlation between semantic distance and rightward bias, whereas right parietal rTMS did not substantially alter a participant's performance. Thus, we documented a “physiological” rightward bias when participants were asked to judge semantic information. These findings suggest that the left parietal cortex contains the neural correlates that underpin the bias in attention and/or mental representation of semantic information.

We suggest that spatial manipulation of semantic material results in the activation of specialised attentional resources located in the left hemisphere. This suggestion is in accord with the “hemispheric activation model” [29], [30] proposing that the distribution of attention in space is biased contralaterally to the more activated hemisphere. We speculated that verbal processing activated the left language-dominant hemisphere more strongly than the right hemisphere. This resulted in attentional shifting attention towards right hemispace.

In line with this recent neuroimaging investigations have implicated a network involving parietal and frontal areas in the orientation of attention in semantic task. Recently, Cristescu et al. [31] showed that semantic orienting selectively engaged activation in the areas of left-hemisphere involved in the semantic analysis of words. These areas represent key nodes in a widely distributed network, which integrates and retrieves semantic knowledge. The multimodal nature of this network enables the formation of selective semantic expectations, and thus the biasing of brain activity by these expectations. This evidence is in accord with our findings. The reported activation of the fronto-parietal network by semantic orienting cues supports the existence of a ubiquitous, general-purpose attentional orienting network. According to the “Spatial Registration Hypothesis” [42], the neural activity mediating language is likely to be modulated by head and eye position, similar to the way in which tactile processing is influenced by head and eye position [46]. Cross-modal (tactile–visual) integration in the posterior parietal cortex may be accompanied by cross-material (spatial–linguistic) integration in the posterior left parietal cortex. In addition, Coslett [42] has suggested that cerebral damage to the parietal cortices impairs contralesional spatial registration, and consequently damages even the activity of non-spatial operations like lexical retrieval and semantic search. This hypothesis is in accord with the clinical reports of the two lesion studies mentioned above. In fact, both the aphasic patient described by Coslett [42] and the agrammatic patient described by Chatterjee et al. [43] had left parietal lesions. This clinical evidence strongly supports the rTMS findings reported here.

Future research is needed to establish whether the effect we documented is specific to semantic processing or can be generalised to language. In particular, future investigations will need to establish whether spatial variables can affect other linguistic components such as phonology and syntax.
